# HIV and gender identity expression among transfeminine people in the Western Cape, South Africa – a thematic analysis of data from the HPTN 071 (PopART) trial

**DOI:** 10.1186/s12889-024-19215-0

**Published:** 2024-07-06

**Authors:** Laing de Villiers, Leslie Swartz, Peter Bock, Janet Seeley, Anne L. Stangl, Virginia Bond, James Hargreaves, Graeme Hoddinott

**Affiliations:** 1 Desmond Tutu TB Centre, Department of Paediatrics and Child Health, Faculty of Medicine and Health Sciences, Stellenbosch University, Cape Town, South Africa; 2 Department of Psychology, Stellenbosch University, Stellenbosch, South Africa; 3 Pivot Collective, Cape Town, South Africa; 4 Department of Global Health and Development, Faculty of Public Health and Policy, London School of Hygiene and Tropical Medicine, London, UK; 5 Department of International Health, Johns Hopkins Bloomberg School of Public Health, Baltimore, MD USA; 6 Hera Solutions, Baltimore, MD USA; 7 Zambart, School of Public Health, University of Zambia, Lusaka, Zambia; 8 Department of Public Health, Environments and Society, Faculty of Public Health and Policy, London School of Hygiene and Tropical Medicine, London, UK; 9 School of Public Health, Faculty of Medicine and Health, The University of Sydney, Sydney, Australia; 10 Group for Research on Infectious Diseases, Department of Human Biological and Translational Medical Sciences, University of Namibia, Windhoek, Namibia

**Keywords:** Transgender, Gender identity, HIV, Gender expression, South Africa

## Abstract

**Introduction:**

Transfeminine people in South Africa have a high HIV risk due to structural, behavioural, and psychosocial factors. Transfeminine people and feminine identifying men who have sex with men (MSM) are often conflated or grouped with transgender or MSM categories in HIV service programming, although they don’t necessarily identify as either. We aimed to investigate gender expression among feminine identifying people who were assigned male at birth. We examined how local conceptualizations of sexuality and gender intersect with the key population label of ‘transgender’ imported into local HIV programming.

**Methods:**

A qualitative cohort nested within the HPTN 071 (PopART) trial included longitudinal, in-depth interviews with eight transfeminine people (four who disclosed as living with HIV). Data were collected approximately every six weeks between January 2016 and October 2017. We used a combination of thematic analysis and case study descriptions to explore gender identification among participants.

**Results:**

Of the eight participants, only one accepted ‘transgender’ as a label, and even she used varying terms at different times to describe her identity. For participants, a feminine identity included dressing in normatively feminine clothes; using feminine terms, pronouns and names; and adopting stereotypically feminine mannerisms. Participants would switch between typically feminine and masculine norms in response to contextual cues and audience. For example, some participants accepted identification as masculine gay men amongst their family members. Among peers, they expressed their identity through typically more effeminate gender characteristics, for example self-identifying as “femgay”. With partners they often also took on a feminine identity role, for example identifying as women in sexual and romantic relationships (meaning they viewed and expressed themselves as the feminine partner in the relationship).

**Conclusions:**

Our findings are amongst the first exploratory and descriptive data of transfeminine people in South Africa. We show how transfeminine people navigate fluid gender identities that could pose a challenge for accessing and utilizing HIV services that are currently set up for transgender individuals or MSM. More work needs to be done to understand and respond to the diverse and shifting ways people experience their gender identities in this high HIV burden context.

## Introduction

Over the past six years, heightened focus has been placed on transgender women in HIV research worldwide [1, 2]. ‘Transgender’ is an umbrella term conventionally used to describe someone who identifies as a different gender than their biological assigned sex at birth [2, 3], which also includes a variety of gender diverse and possibly non-binary individuals with a range of gender expressions and sexual partner preferences. In some contexts, the term ‘transgender’ is predominantly used to describe people who have accessed or intend to access gender transitional health services to shift their biology to reflect their self-identity [4–6]. As such, the term ‘transgender’ is often connected to a fixed gender identity and often measured at one point in time with participants selecting from a single gender identity label, potentially obscuring experiences of gender as fluid [3]. Indeed, the majority of HIV research with the transgender community to date has focused on fixed gender identity [1]. In this manuscript, we use the term transfeminine from this narrow (mis)conception of transgender and focus on identity rather, regardless of transitional health service intentions. Therefore, ‘transfeminine’ in this context refer to people assigned male at birth who identify as women in major parts of their lives but have fluid ways of expressing their gender identity [7].

In HIV service programming, transfeminine people are often categorized as transgender women, gay men or MSM [8]. Conflation of people who have trans and diverse gender identifications happens in HIV research and programming, where transgender women and MSM are two ‘at-risk’ population categories [9, 10]. This is because in HIV research, there has been a focus on risk of transmission associated with different types of sex, e.g., vaginal vs. anal sex, which has meant efforts to collect data (mostly quantitative) influenced categories of sexual orientation and gender identity to be reduced to limited categories as proxies for risk. The conflation of these categories is not only inaccurate but can also pose a harmful risk in that it does not distinguish the unique experiences and sources of risk and vulnerability among transgender individuals, which require specialised interventions.

In a recent study, Brumbaugh-Johnson et al. [11] used a gender theory lens to show that gender expression and disclosure among transgender people is a complex process based on social enactment and circumstances. For example, in the South African, Western Cape coloured community context, taking on hyper-feminized roles by feminine identifying gay men and transfeminine people helped to regulate and counteract predominant hegemonic masculine discourse and behaviour [12]. In other words gay men and trans women in these communities are expected to be “like a woman”, to take on a feminine identity, in order to be accepted into gender prescribed norms of their communities. Sandfort [13] explains: “to be gendered implies far more than being allocated some neutral and taken-for-granted label but is to be subject to a range of regulatory mechanisms and moral prohibitions.” Because gender is done in social interactions, trans people find appropriate ways of feminine and masculine gender expression, according to certain situations and with different people [14] as a way of fitting in, surviving and protecting themselves from being stigmatized [9].

Very little knowledge about the experiences of transfeminine people has been codified into dominant public and research narrative particularly in South Africa, a country that accounts for approximately one fifth of all people living with HIV in the world [15]. We aimed to show the complexity of transfeminine people’s experiences in a high HIV-burden area, the Western Cape of South Africa. Specifically, we (a) describe the different terms participants use to describe their gender identity and gender expression; and (b) present case examples of how gender identity is expressed differently for these women depending on their contexts.

## Methods

### Study design

This was an exploratory study using secondary data from a qualitative cohort. We made use of a combination of thematic analysis and case study descriptions. Data used for this study were accessed from a sub-sample of a qualitative cohort study nested in the HPTN 071 (PopART) trial. As part of the nested social science evaluation, we conducted qualitative research with a cohort of approximately 90 families spread across nine study communities in the Western Cape, South Africa. Eight of these families included at least one transfeminine person (data analyzed here).

### Recruitment

Recruitment for the bigger qualitative study included purposive sampling of specific interest groups that were relevant to larger trial outcomes, including people living with HIV, MSM, trans persons, previously incarcerated persons, sex workers, young people and more [16]. To identify transfeminine people, we approached and had initial conversations about gender and sexual minority people in the community with a variety of community members; clinic staff; trial intervention staff; Lesbian, Gay, Bisexual, Transgender, Queer and Intersex (LGBTQI) community members; and men’s health clinic staff (asking them for referrals of transfeminine people they knew or worked with). People identified as potential participants through these conversations were then approached in person and asked whether they were interested to participate. When we approached potential participants, we explained how we had come to approach them, e.g., I met X who suggested you might be interested in our project. Through this process, thirteen transfeminine people (persons assigned male sex at birth who have feminine gender identity and expression) were initially approached for participation, five of whom were lost to follow up or opted out of participation in the study. Of the remaining eight, four disclosed to us that they were living with HIV.

### Data collection

Based on what Geertz [17] termed as “deep hanging out”, the social and behavioural science team spent time in people’s homes and with them in their daily lives, having a variety of topic-centred discussions. Therefore, data collection was informed by ethnographic and participatory research principles. Data collection with these individuals and their households entailed a series of in-depth interviews and observations structured into “modules” that focused on domains of life (family structure and kinship, mobility, how they get by, love and sexual experiences, engagements with HIV services and care, as well as future aspirations) [16]. The discussion guides were semi-structured and included multiple topic areas within the module with open-ended probing questions. The socio-behavioural science team developed these guides and topics. The number of interviews per participant varied according to their preferences and pragmatic scheduling. The duration of any particular interview ranged from brief, approximately 20-minute check-ins to 3–4 h long interactions as they went about their daily routines. The full study guides are available per request (see section *Availability of data and materials*), but an outline version of the study guide has been previously published with an earlier article from the same project [8].

Data collection was done by pairs of graduate social science researchers. Our team recorded the discussions with voice recorders and semi-structured field notes and took pictures of relevant activities. All interactions with participants happened in situ in study communities – usually in participants’ homes, but also often as they moved about the study community completing their daily activities. Researchers interacted with households for several hours per interaction and multiple times over the course of the data collection period, which took place from January 2016 to October 2017. Apart from recorded interviews, we also collected data via field notes, reflection documents, photographs and participatory research activities (e.g., drawing a map of the community with the participants) during visits. For this manuscript we only made use of the transcripts for the thematic analysis and included field notes and reflection documents to help develop the case descriptions.

The researchers were trained in ethnographic research skills and together as team workshopped data collection processes (doing field visits, writing up of documents and submitting data). Debriefs and reflections were done as the researcher pairs drove home after fieldwork or arrived at the office. In total, discussions and observation activities with the eight participants and their household members took place over 146 h, of which 82 h of interviews were with the transfeminine people. The first author (LdV) was one of the researchers to visit most of the eight participants involved in this analysis and he transcribed a majority of the interview data (other transcriptions finalized by graduate socio-behavioural science assistants who were part of the data collection team but are not authors of this manuscript). Interviews were done in either local languages of Afrikaans or Xhosa and then transcribed and translated into English for analysis purposes.

### Data analysis

We conducted a thematic analysis of the terms and concepts used by participants related to their gender identification, with the core analysis team involving the first (LdV), second (LS) and last author (GH). We followed an open coding process, done with NVivo 12 (QSR International) software, which allowed us to identify different places in transcripts where participants spoke about and used terms in reference to their gender identities. From these initial codes we developed a themed table in Excel, that was refined and is presented in Table 1.

**Table 1:** Gender identity and expression terms used by participants

Pseudonym	Words participant uses to refer to gender identities (all, not just their preferred label)	Situations where participant identifies in feminine ways	Situations where participant identifies in masculine ways
Simone Owen Andrews	skeef^a^ (skew/not straight), moffie^b^ , feminine gay, transgender, gay, vroumens (woman), feminine pronouns, meisie (girl), drag	Around friends and LGBTQI peers; with sexual and romantic partners	With family (sister), extended family (aunts, cousins) heterosexual friends (in these instances others as well as herself use her masculine name, Owen)
Steven Jansen	Daughter/woman, *moffie*, femgay, cross-dresser, woman, man (limited use), more than a woman, drag queen, *meisie* (girl), feminine, “mable”, bottom	Around friends, in social gatherings (clubs & shebeens), in sexual interactions and relationships	With family members
Stacey Benjamin Martins	*meisie* (girl/woman), *moffie*, feminine, femgay, gay, female, woman, bottom	Around friends, social gatherings at clubs and shebeens, in sexual interactions and relationships	In new relationships having to negotiate her biological anatomy, with extended family members and community members
Conry Curella Jenkings	Gay, *meisie* (girl), femgay, queen (as part of the pagent/modelling shows), drag queen, *vroumens* (woman), male, “mable”, feminine, bottom	Cross-dressing and feminine identity in public with peers and friends, cross-dressing at home, in sexual interactions and relationships	On formal documentation and accessing services: “*we don’t ever say we’re males (laughs) unless you want to really know or you have my ID in hand*”, at school, initial and potential partners (having to explain assigned sex at birth)
Girlie Gregory Jones	*meisie* (girl), man, woman, gay	In sexual interactions and relationships (with her partner)	In romantic or sexual relationships – sometimes needing to be tough to counteract violence, on formal documentation, with her family
Georgina George Samuels	Woman, *moffie*, feminine pronouns, *“vrou”* (woman), man	Dresses and express feminine with family, family who acknowledges she has feminine hormones, in sexual interactions and relationships	Outside of regular social circles: clinics, work, shops, community; family also uses masculine name and pronouns to refer to her; with masculine/male friends
Sizwe Thafeni	Gay, *moffie*, MSM, lady/woman, male (but does not identify with the term), bottom	In sexual interactions and relationships; at work	Formal documentation (showing her ID document), with family members (like her mother)
Patricia Arends	Guy/man, woman, gay, *moffie*, *meisie* (girl)	In sexual interactions and relationships	Formal documentation (on ID document), community members, friends and household members

^a^Italicised words are Afrikaans

^b^The term “moffie” is a derogatory Afrikaans term used to refer to effeminate gay men, with possible origins from the Dutch “mofrodiet” for “hermaphrodite” [20], [21]. Most of the participants used the word as a self-claimed term to refer to themselves and their friends and peers

We then provided case descriptions for the different participants to show negotiation of gender identity terms in different contexts. We initially developed larger narratives about each participant – including sub-sections about their house (physical) and household membership structure, employment, social and other life experiences. These larger narratives synthesize data across multiple interviews per participant and using other data sources, similar to a narrative analysis technique used by Riessman [18]. From the larger narratives, short case descriptions were then developed to show how gendered terms and identifications were dealt with by participants on a daily and lived basis and how these terms and identities possibly influenced their HIV care. The case descriptions were used to contextualise the gender identity terms used by participants and were purposively designed and added by the analysis team.

### Ethical considerations

We would often do extended visits with the participants, walking around in their community, accompanying them to clinic visits and modelling/drag show events. Due to the sensitive nature of the work and spending time in people’s personal lives, we found that the longitudinal nature of the engagement helped build rapport between researchers and participants. In addition, the strong connections developed with participants allowed us to manage the discussion of events in their lives which were traumatic, and might re-invoke trauma, sensitively.

All participant household members signed consent forms to have their households participate in the qualitative cohort. The consent also expressed research outside of the households and documenting various other parts of the participants’ lived experiences. For any events and activities researchers ensured participant willingness. Each household member also had a personal choice to participate or not. Participants were informed during the consent process about the possible sensitive nature of the interviews. Pseudonyms are used here for each participant, family members, neighborhoods and towns to protect confidentiality. Participants were not given incentives. Instead, research staff received allowances of approximately 80 South African Rands (about 6 USD) per visit to share household costs, contribute to shared meals, and/or gifts for participants and their households.

## Findings

For participants, expressing their ‘feminine identity’ included dressing in normatively feminine clothes, using feminine terms, pronouns and names, as well as other feminine attributes and mannerisms. Participants’ gender identity and expression changed often in the way they referred to themselves and how others referred to them in different contexts. We first provide a descriptive table of terms that participants and people used throughout the study for referring to their gender identity. We then present case descriptions as examples to illustrate how gender was navigated by different participants in different contexts. The pronouns used in the case descriptions were the chosen pronouns that participants used to refer to themselves.

### Participant demographics

Seven of the eight participants were Coloured identifying Afrikaans-speaking, with one who was a Black African Xhosa-speaking person. ‘Coloured’ is a loaded racial and cultural category with an Apartheid historic lens that grouped together a wide variety of ethnic groups of people, including people who are of mixed racial descent, descendants of early Khoi/San inhabitants of South Africa as well as descendants of people who were imported as slaves from Eastern communities. It used here as a social identity. Participants’ ages ranged between 21 to 32 and they all were from lower income settings, with average household earnings that were between 100 USD to 600 USD per month. Because participants were from largely homogenous racial communities (largely due to South African communities’ inherited Apartheid racially imposed spatial division), we didn’t find any race-based nuances in participant’s gender and sexual identity experiences.”

### Description of terms participants use to describe their gender identity and gender expression

Participants used a variety of gender identity expressions and ways of referring to themselves. These terms and gender expressions were, however, context bound, referring to people, time and place. Table 1 shows the terms (Italicised words are Afrikaans) used by participants as well as situations where their gender identity might be expressed in a more masculine and feminine manner.

The table illustrates pseudonym birth given names of the participants (which relate to their masculine biological assigned sex at birth) and then some participants used self-assigned names and nicknames connected to their feminine identity. All names here are fictional pseudonyms that illustrate this pattern. For example, Simone’s birth given name is Owen Andrews, but she has chosen the name Simone as it more accurately connects to her feminine gender identity. Alongside gender-typical names, we show a variety of terms used by participants throughout the study in their interviews. These are terms that others might use to refer to them or ones that they use to refer to themselves. The term “moffie” for example is a derogatory Afrikaans term used to refer to effeminate gay men, with possible origins from the Dutch “mofrodiet” for ‘hermaphrodite’ [19, 20]. Most of the participants used the word as a self-claimed term to refer to themselves and their friends and peers. ‘Moffie’ is also often used by community members to address the transfeminine people we interviewed and their peers. Most of the terms used by participants are feminine identifying terms, like femgay, queen, woman or feminine, but they also sometimes used terms related to their biological assigned male sex at birth like man, guy and male. Some terms also had particular reference to their sexual orientation or sex role, like gay or bottom (referring to their preference as receptive anal sex partners). The table also portrays situations where participants used either feminine or masculine identities depending on the situation. For example, Simone explained that she used feminine gender identity expression terms amongst transfeminine and gay peers and with her romantic partners, whereas her family members (e.g. sister, cousins and aunts) typically referred to her in a masculine way by using her birth given name, Owen.

### Case examples of how gender identity was expressed differently for these women depending on their contexts

24-year-old Simone Owen Andrews is from Town D, a mixed metro and low-income community in the Western Cape. Simone is living with HIV and identifies with the term “transgender woman”, however she also uses the term “gay”. Growing up she always felt and behaved in a feminine manner, playing mostly with girls, cross-dressing, and in this way of being, she told us that people could distinguish that she was not a heterosexual boy. She often uses the term ‘gay’ to refer to herself. Some people still call her by her masculine birth name, Owen, which could potentially mean her having to negotiate a masculine component of her identity (that in some ways she is still seen as a man by some people). Below is an excerpt from an interview in April 2017 where she uses a ‘gay’ identity term:

Researcher: okay but then how do you see yourself? You know if they talk about LGBT- or a range of categories, what, how do you categorise yourself?

Simone: for now it is “gay”

Researcher: just gay?

Simone: yes

For Simone and most of the study participants, their feminine gender identity was present from a young age and they have seen it develop over time as they started dressing in feminine clothes, participating in drag/modelling shows and describing themselves as a woman (the feminine identifying partner) in their romantic and sexual relationships.

Stacey (born Benjamin Martins) is a 29-year-old self-identified ‘femgay’ individual also living with HIV. She lives in an informal community at the outskirts of a winelands farming town in the Western Cape. Stacey does not live with her immediate family. She lives a very mobile/transient lifestyle, moving between different houses in the immediate neighbourhood and relying on extended family members, friends and other femgay peers to support her with a place to stay, food and other necessities. Even though Stacey might fall under the transgender umbrella, she actually took offence when in an early interview the interviewer mistakenly labelled her gender identification as transgender, as seen in the excerpt below in June 2016:

Researcher: We have a lot of things we are interested in, in sex and love and like how many partners people have had and like where does a person meet, like that’s very interesting for me, especially if a person’s transgender, like

 (Stacey interrupts her)

Stacey: Me? transgender? (As her voice squeaked in disbelief and she seems to be taken aback).

 (Researcher tries to regather her words, but Stacey continues)

 I’m femgay (she proudly responds).

Curella (Conry Jenkings), a 28-year-old transfeminine person, also identifies as ‘femgay ‘. She lives outside the Cape Town metropole with her mother, sister and her mother’s boyfriend. Her family and extended family, living with her, address her as Conry and it’s also how she refers to herself. However, dressing and being a woman is how she feels most comfortable and one of the places that she gets to be her full, cross-dressing and feminine self is at cross-dressing modelling shows. Her modelling and feminine name is Curella. She explained her ‘femgay’ identity below (August 2016):

Researcher: what is a drag queen?

Conry: a drag queen is similar to us in that they also do shows (pause) like put on dresses, and like heels and stuff like that (pause) and then you get

Researcher: but is a drag queen necessarily someone who is gay?

Conry: yes … and you have them, they are gay but he doesn’t put on women’s clothes, he wears normal men’s clothes

Researcher: and so you would distinguish between the two?

Conry: yes you get uhm femgay and then you get (drag queens)

Steven Jansen lives in a small wooden house, which is in the backyard of her aunt’s house, with her mother, father and sister, who all view her as a gay man. Steven, like most of the women we interviewed, used a variety of terms to refer to her gender and sexual identity, including gay, femgay and ‘moffie’. Steven has also navigated her gender identity from a young age and even though she is more assured of her feminine gender identity, it still takes navigation in different spaces as she explains about going out socialising at night:

Now a lot of them have made the excuse that “you are more of a woman than … women really are”. That is their excuse … like last night I was sitting in company … then a guy brought me water and I told him, “ah you are too sweet”, and gave him a kiss on the cheek and then he told me, “Jesus!” and his friend said, “God, you do everything just like a real woman, just like a real woman is supposed to do”.

(Steven, May 2017)

Sizwe identifies as MSM but uses feminine pronouns and terms to refer to herself. For Sizwe, her gender identity expression, or the way she has to navigate it, is also influenced by cultural significance associated with spaces, as she is expected as a biological male to go through traditional rites of passage and circumcision into manhood. She explained in a later interview that if she were to go through initiation that it wouldn’t be for herself. She would do it purely for cultural obedience. She explicitly stated that she wouldn’t comply with some of the post-initiation observances, like dressing in a jacket and hat. Similarly, when men get together for traditional ceremonies they will sit together in a fenced-off area called a “kraal”. Below is her reaction to the researcher in explaining her gender role amongst men in the *kraal*:

Researcher: okay and then now when you have to sit in the *kraal* will you sit then with other men maybe?

Sizwe: if I am going to sit, I will arrive in the *kraal*, my dear, but otherwise I won’t sit long. I won’t continuously … what do I want in the *kraal*?

Researcher: it’s a man among men there

Sizwe: I am so weak (hinting at her feminine traits)

Researcher: you would have also been a man according to culture

Sizwe: you can see I am weak … oh no I am not a man you see me dear

(November 2016)

Girlie Gregory Jones and Patricia Arends are two transfeminine people who do sex work. Sex work for them is not a choice, but something they were both coerced into and stayed in as a means of survival. Their navigation of their gender identities was fraught with even more challenges and problems as they have to deal with coercive gangsters who force them into sex work, as well as clients with whom they have to navigate their gender identity.

Below, Girlie expresses the confusion with which she doesn’t actively seem to have to consider whether she is a man or woman; but the realisation that she has aspects of both, even though she identifies more as a woman:

I just am the way I am, see? I am a man but I like dressing like this … I know I am a man but I feel like a woman, do you see? … I just am like this.

(August 2017)

## Discussion

We found that transfeminine people use a variety of ways to express their gender identity. Although all the participants identified as women and in a feminine way, they used a variety of feminine and masculine terms/concepts to refer to themselves in different contexts, depending on spaces/places, time and who they were with. This variety was reflected in interactions in their personal lives, amongst peers, friends, family, and partners, as well as in social interactions with community members, and when accessing services and other formal processes where identification documentation is requested or required. Our findings suggest ways of understanding local gender terms from participants’ individual lived experiences and how it relates to more global understandings of transfeminine persons.

Globally, trans people may use a range of local and informal gender identity expressions like ‘fairies’, ‘queens’, ‘feminine gay’, cross-dressers and transsexuals to refer to themselves [4, 20], and which in other contexts or used by others can be derogatory slurs. In different parts of the world, so-called feminine males have a range of gender identifications embedded in culture and language: *bakla* or *kathoey* in Thailand, *Travesti* in central- and South America, *Hijras* in India and *Waria* in Indonesia for example [2, 22]. A special issue of Global Public Health in 2016 emphasized a renewed call for a balanced understanding of sexual and gender diversities aimed toward re-evaluating approaches to prevention and health promotion [10]. Local South African researchers have identified similar gaps in HIV research, as they acknowledge the need for deeper investigation into diversity, fluidity and complexity of gender expressions among MSM and transgender women especially when it comes to HIV risk and service access [23–25].

It is evident that gender expression is contextual to the lived reality of a transgender person. As Brumbaugh-Johnson [12] found, transgender people’s expression and disclosure of their gender identity is navigated by expectations and reactions of others, particularly in avoiding potential harmful situations. Trans people for example in the UK and Portugal self-regulated their gender expression and avoided specific spaces perceived as unsafe [14]. In particular, transgender people’s gender identity displays and expression need to be contextualized in time and space [15], similar to findings we found in our study. Recent research has shown how public and private spaces are navigated by trans people to find safe space for them to openly express their gender identity and avoid more stigmatizing ones where they may experience discrimination [26–28]. The private home space is not necessarily more or less of a vulnerable space for trans people than the public and communal space as, for example, certain areas in the community might be avoided and other not, especially at certain times. Or that during certain times a trans person might feel less accepted at home if there is a family member who visits who has trans- or homophobic beliefs.

Participants in our study similarly used a range of terms that were contextualised in time and space. For example, participants wouldn’t use the term gay to identify themselves if their family and community members didn’t use the term, or they would not have adopted an originally denigrating term like “moffie”, if it wasn’t freely used to criticize and discriminate against them. The way that transfeminine people navigate use of terms for gender identity is, as we’ve seen with a variety of global indigenous and locally used terms, very important to how they potentially access health services and other services that require the use of their identification document. Previous research, similar to our results, have pointed to how trans people had to navigate their gender identity in private and public institutions, like home affairs, the workplace, banks, schools and more, where they often faced unequal treatment, the creation of various obstacles and outright exclusion from these spaces [14].

In a range of recent South African studies, HIV researchers have been interested in the liminal group of women who fit into the transgender category, like feminine-identifying MSM [29, 30], gender non-confirming MSM [32–33], non-binary and trans-women [34]. Some terms or ways of identifying include “cross-dressing”, “feminine identifying”, “drag queen”, “being trapped in men’s bodies”, and “gay women” [24, 25, 35]. A study by Sandfort et al. [35] showed a variety of colloquial terms used by feminine identifying MSM and transfeminine people in the Tshwane province of South Africa, including: *Stabane, stuzana* or *gemmi* (for gay men, demeaning); *trasi*, *skezonke* (men in the wrong body, persons having a penis and a vagina); *Maho, magene* (men who are bottoms); *moffie, watermuis, tarashushu* (feminine gay men).

We show in a recent publication, where we used the same sample and data, that gender identity was often conflated with sexual identity for these transfeminine people in the Western Cape, South African context [8]. In this conflation of gender and sexual identity from other people towards transfeminine people, stigma intersected with other social identities like being a sex worker, using drugs, living with HIV and more [8]. In that study we found that this complicated the way these transfeminine people accessed HIV services (including testing, treatment, and adherence support), since these spaces brought up anticipation and experiences relating to their stigmatized identities.”

Despite some progress to further HIV research among transgender populations globally, very little research on the topic has been done in South Africa [36]. Our results offer initial understandings of the complexity of how transfeminine people navigate their gender identity in different contexts and allude to how this is relevant to HIV service access. From our findings we recommend: gender transformative research; interventions and training that approach varied gender identity terms and expressions; pronouns or sexual orientation preference to be included on clinic forms and research variable categories offered as ways for healthcare workers to engage with patients [37]. For research interventions the distinction between gender identity and sexual orientation should allow participants to choose from a variety of categories or even allow self-identifying themselves on surveys and questionnaires. At facility level, trainings can allow staff to similarly understand the fluid nature of gender and sexual identities of trans people and support trans people to openly express their gender identity and how it may change in different contexts, as well as discussing their unique biological risks and behaviours related to their sexual orientation.

From the local terms and concepts from other global studies (Hjira, Travesti, two-spirited and other transgender groups) [2, 6], we suggest that similar exploratory work is urgently needed to better understand South African sexual and gender minority individuals, in particular transfeminine people, and answer questions such as: How do transgender identities intersect with HIV service access? Could transgender-specialized services potentially exclude transfeminine people who don’t use trans language and who have no interest in gender reassignment, hormonal and other transitional treatment and information? What are some of the other gendered terms used by transfeminine and feminine identifying MSM and gay men in South Africa, specifically colloquial terms in participants’ local languages? These are some of the questions we think nuance how health services and programs for transgender people can be better developed and be supportive of local concepts in the South African context.

For our analysis and reporting on our findings we considered trustworthiness of specifically studies with qualitative data, as it involves establishing credibility, transferability, dependability and confirmability [38]. This paper also conforms to majority of the COREQ criteria for reporting on qualitative research for interviews and focus groups [39]. Therefore, the strength of our study is supported by the rich data we collected, using a wide range of data collection methods, about the lived experiences of participants, which enabled us to understand the layers of stigma and trauma transfeminine people experience, how they cope with these stresses, and how they view their gender identity. In addition, the longitudinal nature of the data allowed us to confirm information about especially sensitive topics that take time to unpack as trust was built in the research relationship. The longitudinal nature of the data therefore helped in particular with the credibility of our study. Transferability was supported by the thick description of cultural and social relationships as unpacked in this study [38].

Limitations to extrapolation from our findings include: Firstly, our sample size was small. Secondly, the people identified as transfeminine in our study were difficult to find, interview, and retain. Often, transfeminine participants were socially marginalised, moving around a lot and experiencing many interruptions to their daily routines. This may mean that the experiences of participants retained in the study may not be representative of all transfeminine people in the area (e.g., five participants were lost to follow-up). However, our study draws strength from being the first exploratory study of transfeminine people in the Western Cape. In terms of trustworthiness of our study, we have shown limited confirmability and dependability by not having done a proper audit of our neutrality in the study, which is particularly tricky because the data used needs to be applied for (so we can’t share it openly). We have tried to be as reflexive as possible by showing our results clearly in the presence of current research [38]. A final limitation to the study was that the authors (and the other data collectors) did not self-identify as transfeminine and could misunderstand and misrepresent the participants’ experiences because of this.

## Conclusions

In this study we found a variety of terms that South African transfeminine people use to describe their gender identity. These terms and concepts are largely contextually bound and transfeminine people in the study, even though all eight participants identified in a feminine way, often used a variety of feminine and masculine gender identifying terms depending on different contexts. These insights can be used by HIV service providers to better meet the needs of this unique population. In the context of HIV service access, this means asking about and accepting people’s gender identity and expression, instead of assuming or imposing a category, like ‘transgender’, and tailoring service delivery to the unique needs of gender fluid populations. In addition, our findings will help to inform the development of gender-responsive HIV prevention, care and treatment services to support countries in achieving the societal enabler targets and global HIV goals by 2030 [40].

## Data Availability

The data used for this manuscript form part of a databank for the HPTN071 (PopART) study, which can be made available by application and request to the trial working group. Persons to contact regarding this are co-authors PB (in-country co-PI) and GH (socio-behavioural science lead and qualitive cohort design).

## Abbreviations

HIV: Human Immunodeficiency Virus
MSM: Men who sex with men
PopART: Population Effects of Antiretroviral Therapy to Reduce HIV Transmission (PopART): A cluster-randomized trial of the impact of a combination prevention package on population-level HIV incidence in Zambia and South Africa
LGBTQI: Lesbian, Gay, Bisexual, Transgender, Queer and Intersex
